# Plasma homocysteine and B vitamins levels in Nigerian children with nephrotic syndrome

**DOI:** 10.11604/pamj.2014.18.107.3678

**Published:** 2014-06-02

**Authors:** Bose Etaniamhe Orimadegun, Adebola Emmanuel Orimadegun, Adebowale Dele Ademola, Emmanuel Oluyemi Agbedana

**Affiliations:** 1 Department of Chemical Pathology, College of Medicine, University of Ibadan, Nigeria; 2 Institute of Child Health, College of Medicine, University of Ibadan, Nigeria; 3 Department of Paediatrics, College of Medicine, University of Ibadan, Nigeria

**Keywords:** Nephrotic syndrome, homocysteine, cardiovascular risk, vitamins, folate, children

## Abstract

**Introduction:**

Available data on plasma homocysteine level in patients with nephrotic syndrome (NS) are controversial with increased, decreased and unchanged values reported. Therefore, plasma homocysteine and serum B vitamins in Nigerian children with NS were assessed in this study

**Methods:**

Fasting blood samples were analysed for plasma homocysteine, serum folate and B vitamins in 42 children with NS and 42 age and sex-matched healthy controls in this case control study. Data were compared between NS and control using t test and Chi square. Relationships were tested with regression analysis with p set at 0.05.

**Results:**

Prevalence of hyperhomocysteinaemia, low folate and cyanocobalamin in NS was 57.1%, 14.3% and 9.5% respectively. The mean homocysteine level was significantly higher in NS than control (11.3±2.6µmol/L versus 5.5±2.3µmol/L). Also, NS had lower folate and cyanocobalamin than control: 9.1±3.9ng/mL versus 11.2±3.1ng/dL and 268.5±95.7pg/mL versus 316±117.2pg/mL respectively. Weak but significant correlation between homocysteine and serum albumin (r = 0.347), folate (r = -0.607) and vitamin B12 (r = -0.185) were found in the NS group. Significant relationship was also found between homocysteine and vitamin B12 (ß = -0.64, 95% CI = -1.20, -0.08) after controlling for folate and vitamin B6 levels.

**Conclusion:**

Clinically important hyperhomocysteinaemia and low B vitamins occur in Nigerian children with nephrotic syndrome. This data suggest that potential usefulness of folate and vitamin B supplementation for reducing high homocysteine levels in nephrotic syndrome need to be further investigated

## Introduction

Nephrotic syndrome (NS) is a disease of the kidneys characterised by the presence of hypercholesterolemia, massive proteinuria, and hypoalbuminaemia with or without oedema [1]. It is primarily a paediatric disorder and it is 15 times more common in children than adults. Worldwide, the estimated annual incidence of NS is 20 to 70 cases per million children and 3 cases per million adults [1]. In Nigeria, the prevalence of NS varies from about 1.3% to 14.6% among hospitalised children with kidney diseases with the peak age of 1-6 years [2, 3]. NS is twice more common in boys than in girls in a typical Nigerian hospital setting [4]. The reason for the gender difference in the incidence of nephrotic syndrome is not clear in literature. However, the disease can present with serious morbidity patterns that make outcomes unpredictable.

Homocysteine, a thiol-containing amino acid is produced by intracellular demethylation of dietary methionine [5] which can either be remethylated forming methionine or catabolised to form cystathionine and cysteine. The remethylation reaction is dependent on the cofactor activity of vitamin B12 and folate [6]; thus, deficiencies of folate, vitamin B12 and sometimes vitamin B6 result in hyperhomocysteinaemia [7]. Reduced and oxidized forms of homocysteine are present in plasma, and their fasting plasma concentrations denoted as total homocysteine, are a reflection of intracellular metabolism and cellular export of homocysteine [5]. In many diseases, high plasma levels of homocysteine have been attributed to deficiencies of B vitamins, which may be worsened by inherited abnormalities of the enzymes involved in its metabolism [8]. Renal function influences plasma homocysteine levels. Thus high levels of homocysteine have been described in patients with some renal diseases [9–11].

Though high level of plasma homocysteine is an independent predictor of cardiovascular complications and poor outcomes of many diseases [12, 13], reports on its prevalence and relationship with other biochemical parameters of children with NS are still being controversial discussed. Three earlier reports showed that plasma homocysteine levels may be decreased [14], equal [15] or increased [16] in NS compared to patients without NS with similar renal function [14, 15] or to healthy controls [16]. A better understanding of these relationships may advance current knowledge of renal physiologic processes, as well as efforts towards finding an effective therapy. Also, understanding of the correlation of folate, vitamins B6 and B12 supplementation on plasma homocysteine in children suffering from NS may place scientists and physicians in better position to make informed treatment decisions and policy. Therefore, this study was carried out to determine relationships of circulating levels of homocysteine, folate, vitamin B6 and vitamin B12 in patients with NS among Nigerian children.

## Methods

**Study site and population:** This study was carried out at the Paediatric Nephrology Clinic and children's wards of the University College Hospital (UCH), Ibadan. The Hospital is located in Ibadan North Local Government Area, Ibadan (IBNLGA). IBNLGA is one of the five Local government areas that constitute the main Ibadan. The University College Hospital, Ibadan serves as the main renal centre for the residents of Ibadan and the people of the South-Western region of Nigeria. The people living in this region are mainly of the Yoruba ethnic group. Children aged less than 18 years are the population of interest for this study. According to 2006 Census, they constitute about 15 – 20% of the population of Nigeria.

**Study design and participants:** This was an age-sex matched case control study. Cases were children who presented with symptoms, signs and laboratory features of NS, while the control group comprised apparently healthy children, who came for routine check-up or minor surgeries at the clinics without symptoms suggestive of renal diseases. Diagnosis of NS was based on the presence of massive proteinuria (24 hour urinary protein > 40mg/m^2^/hour or >50mg/kg/day or proteinuria on dipstick urinalysis of 3 pluses (+++), hypoalbuminaemia (serum albumin < 2.5g/dl) and hypercholesterolemia (>220mg/dl). Patients who had features suggestive of liver disorders, severe protein energy malnutrition and those on vitamins supplementation were excluded.

**Sample size calculation:** Joven et al., [16] reported that 26.0% of the patients with NS and 7.4% of healthy control subjects had hyperhomocysteinaemia. Substituting these values in formula for calculating sample size for comparisons of two proportions, a minimum of 16 cases and 16 controls were required for the study to achieve power of 80% at 95% level of confidence.

**Data collection and laboratory tests:** Parents of all eligible children were approached for consent to participate in the study at first visit. Children whose parents gave consents were interviewed by the investigator using a structured questionnaire that contained items on demographic characteristics of each subject. Blood and urine samples were collected for estimation of serum albumin and urinary protein and creatinine. About 5mL of blood sample was collected and dispensed into EDTA containing and plain bottles. Plasma homocysteine level was determined using an enzyme immunoassay method described by Frantzen et al [17] using commercially available kit from Axis-Shield Diagnostics Ltd, UK. Serum folate, pyridoxine (B6) and cyanocobalamin (B12) were determined using high performance liquid chromatography and serum creatinine with fixed-time Jaffe method. Creatinine clearance, Estimated glomerular filtration rate (eGFR) in mL/min was calculated using the following equation: ((140 – age) x Body Weight) / (Serum creatinine x 72) (x 0.85 for females) [18]. eGFR was classified as mild reduction (60-89 mL/min/1.73m^2^), moderate reduction (15 - 59 mL/min/1.73 m^2^), and normal (=90 mL/min/1.73 m^2^) [19].

**Data management and analyses:** Socioeconomic index scores were awarded to each child, based on the parents’ occupations and educational attainment or their substitutes as described by Oyedeji [20]. In this study, average score of one was regarded as upper class, scores of 2 and 3 as middle class, while scores of 4 as lower class. Data were analysed using Statistical Package for Social Scientists (SPSS) 17.0 for Windows (SPSS Inc., IL, USA). All continuous variables were checked for normality using the Shapiro-Wilks test and those found to be non-parametric in distribution were identified. Unpaired two tailed Student t test was used to compare means and detect significant differences in parameters between cases and control. Spearman Rho correlation analysis method was used to assess correlations between variables because of the non-parametric nature of most of the measured parameters. The distribution of low levels of vitamin B6, vitamin B12 and high level of homocysteine was assessed by dividing vitamin levels into quartiles. The relationship of homocysteine with folate and vitamins among the NS group was examined using multivariate analysis. P values lower than 0.05 were considered statistically significant.

**Ethical consideration:** Participation in the study was completely voluntary and based on written informed consent. Participants were made to understand that they were free to withdraw their consent at any time and they would not be denied their due treatments according to the UCH protocol. Ethical approval for this study was obtained from University of Ibadan/University College Hospital Ibadan Ethical Review Committee. Verbal accent was obtained from children older than 5 years.

## Results

### Characteristics of study subjects

Demographic, body mass index and laboratory parameters of study participants were as in Table 1. There were 27 (64.3%) males and 15 (35.7%) females among NS and controls respectively. There was no significant difference in the mean ages of NS (103.5 ± 32.7 months) and control (100.9 ± 29.4 months); p = 0.740. Also, the distribution of participants by parents’ socioeconomic status among cases and controls was not different as shown in Table 1 (p=0.744). Mean Body Mass Index (BMI) of the NS (17.3 ±1.6kg/m^2^) was significantly higher than the control (15.9 ±1.2kg/m^2^); p<0.001. Mean eGFR in the NS was not significantly different from the respective value in the control. On the other hand, the mean total serum protein and albumin concentrations in the NS group were significantly lower than the corresponding values in the control. Moreover, mean total homocysteine of 11.2±2.8 µmol/L in the NS group was significantly higher than the control (5.5±2.3 µmol/L); p<0.001. Mean serum folate and vitamin B12 concentrations in the NS were significantly lower than corresponding values in the control. Conversely, the slightly lower mean vitamin B6 concentration in the NS when compared with control value was not statistically significant.

**Table 1:** Demographic, anthropometric, and laboratory variables of the study population

	NS (n = 42)	Controls (n = 42)	p
**Gender (male/female)**	27/15	27/15	-
**Mean age (months)**	103.5±32.7	100.9±29.4	0.740
**Socioeconomic class (%)**			
**High**	4 (9.5)	4 (9.5)	0.744
**Middle**	26 (61.9)	23 (54.8)	
**Low**	12 (28.6)	15 (35.7)	
**Body mass index (kg/m** ^**2**^ **)**	17.3±1.6	15.9±1.2	<0.001
**Estimated GFR (mL/min/1.73m** ^**2**^ **)**	78.0±33.9	86.5±38.4	0.284
**Serum albumin (g/dl)**	2.2±1.1	4.5±0.5	<0.001
**Total serum protein (g/dl)**	4.9±1.1	6.5±0.6	<0.001
**homocysteine (µmol/L)**	11.2 ± 2.8	5.5 ± 2.3	<0.001
**Folate (ng/mL)**	9.1 ± 3.9	11.2 ± 3.1	0.009
**Vitamin B**_**6**_ **(nmol/L)**	72.4 ± 13.1	75.8 ± 15.2	0.284
**Vitamin B**_**12**_ **(pg/mL)**	268.5 ± 95.7	316.4 ± 117.2	0.043

Variables are reported as mean ± SD except gender and socioeconomic class; NS – Nephrotic syndrome

### Prevalence of hyperhomocysteinaemia and low vitamins

Twenty-four children in the NS group and only three among the controls had hyperhomocysteinaemia giving the prevalence of hyperhomocysteinaemia as 57.1% and 7.1% respectively (Table 2). The NS was 17 times more likely to have hyperhomocysteinaemia compared with the control group. Six (14.3%) children with NS and none of the controls had low serum folate concentration. Four (9.5%) participants in each of the NS and control groups had low vitamin B12 concentration.

**Table 2:** Prevalence of hyperhomocysteinaemia, low serum folate, low vitamin B6 and low vitamin B12

	NS (n= 42)	Controls (n= 42)	P	OR (95% CI)
Hyperhomocysteinaemia (>10µmol/l)	24 (57.1%)	3 (7.1%)	<0.001	17.3 (4.6, 65.1)
Low folate (<3.4ng/mL)	6 (14.3)	0 (0.0)	0.026	-
Low vitamin B6 (<20.0nmol/l)	0	0	-	-
Low vitamin B12 (<133pg/mL)	4 (9.5%)	4 (9.5%)	1.000	-

NS – Nephrotic syndrome

### Levels of plasma homocysteine and eGFR

The distribution of subjects by level of homocysteine and eGFR were as shown in Table 3. In the NS group, 84.6% of those who had moderate–severe reduction in eGFR also had hyperhomocysteinaemia with significant increased risk compared with those whose eGFR was =90 mL/min/1.73 m^2^ (OR = 12.38, 95% CI = 1.83, 83.77). A higher proportion of the NS children who had mild reduction in eGFR (56.3%) compared with those whose eGFR =90 mL/min/1.73 m^2^ (30.7%) had hyperhomocysteinaemia, though the difference was not significant (p = 0.318). Among the control group, 33.3% of those who had moderate-severe reduction in eGFR had hyperhomocysteinaemia, while only 9.1% of those with mild reduction in eGFR had hyperhomocysteinaemia.

**Table 3:** Distribution of subjects by level of homocysteine and reduction in GFR

	tHcy >10.0µmol/L n (%)	tHcy ≤10.0µmol/L n (%)	P	OR	95% CI
**GFR in NS (mL/min/1.73m** ^**2**^ **)**					
15-59	11 (84.6)	2 (15.4)	0.015*	12.38	1.83, 83.77
60-89	9 (56.3)	7 (43.7)	0.318*	3.89	0.62, 13.46
≥90	4 (30.7)	9 (69.3)	1	-	-
**GFR in control (mL/min/1.73m** ^**2**^ **)**					
15-59	2 (33.3)	4 (66.7)	0.514*	5.00	0.35, 38.91
60-89	1 (9.1)	10 (90.9)	1	-	-
≥90	0 (0.0)	25 (100.0)	0.032*	-	-

* Fisher's exact test used, 15 - 59 mL/min/1.73 m^2^: Moderate – severe reduction in GFR; 60-89 mL/min/1.73 m^2^: Mild reduction in GFR; ≥90 mL/min/1.73 m^2^: normal

tHcy – total homocysteine

### Relationship of plasma homocysteine with age, vitamins, albumin and GFR

Correlations between plasma homocysteine level and each of age, BMI, serum folate, vitamin B6, vitamin B12, albumin, eGFR in NS patients and control were as shown in Table 4. There were significant correlations between plasma homocysteine level and each of serum albumin (r = 0.347, p = 0.024), eGFR (r = -0.844, p = 0.020), serum folate (r = -0.607, p = 0.027) and vitamin B12 (r = -0.185, p = 0.045) among the NS group. In the control group, homocysteine significantly correlated with serum albumin (r = 0.566, p = 0.029), serum creatinine (r = -0.330, p = 0.035) and eGFR (r = 0.32, p = 0.037).

**Table 4:** Correlation of homocysteine with age, serum folate, vitamin B6, vitamin B12, albumin, estimated GFR in NS patients and control at baseline

	NS patients		Control	
	r	P	r	P
Age in years	-0.189	0.237	-0.154	0.336
BMI	0.272	0.082	0.156	0.322
Serum albumin	0.347	0.024	0.566	0.029
Creatinine	-0.336	0.039	-0.330	0.035
Estimated GFR	-0.844	0.020	0.323	0.037
Folate	-0.607	0.027	-0.111	0.416
Vitamin B6	0.084	0.597	-0.292	0.061
Vitamin B12	-0.185	0.045	-0.253	0.106

Note: Spearman rho correlation was done because of the non-parametric nature of many of the variables

Since most of the variables that were significantly different between the cases and control by univariate analysis could influence the circulating levels of vitamin B6, vitamin B12 and homocysteine, these differences were tested in a multivariate analysis (Table 5). A statistically significant independent relationship was found between homocysteine and vitamin B12 (ß = -0.64, 95% CI = -1.20, -0.08) after controlling for serum level of folate and vitamin B6.

**Table 5:** Multivariate analysis showing relationship of plasma homocysteine with folate and vitamins among the NS group

	β	95% CI of β	P
**Vitamin B12 (pg/mL)**	-0.64	-1.20, -0.08	0.027
**Vitamin B6 (nmol/L)**	0.84	-0.40, 2.09	0.180
**Folate (ng/mL)**	-0.17	-0.39, 0.05	0.127
**Constant**	0.95	-0.25, 2.14	0.119

## Discussion

This study provides information on the impact of nephrotic syndrome (NS) on homocysteine and vitamins levels in the Nigeria population which was not found in literature before the study. The data in this study have shown that Nigerian children with the NS had high plasma levels of homocysteine, low serum levels of folate and vitamin B12 more frequently than healthy individuals. There are controversies about the prevalence of hyperhomocysteine among NS in previous studies. These studies showed that plasma homocysteine levels were either decreased [14], equal [15] or increased [16] in NS patients compared to patients without NS and of similar renal function [14, 15] or to healthy controls [16]. The finding of higher level of homocysteine in NS in the present study therefore agree with the report of three previous studies [16, 21, 22] but contradict others [14, 15]. Previous studies have shown that plasma tHcy concentrations differ among ethnic groups [23] therefore the possible reason for the differences might be the difference in the population, race or ethnicity of study participants. The significant hyperhomocysteinaemia observed in the NS children could be due to either a block in homocysteine remethylation or a disturbance in cysteine disposal. Suliman et al., [24] demonstrated that homocysteine-lowering regimens in chronic kidney disease needs to always include folate in a clinical trials. It was proposed that a block in the decarboxylation of cystinesulphinic acid, the intermediate between cysteine and taurine, could be an important determinant of the hyperhomocysteinaemia commonly encouraged in patients with renal diseases[24]. In the present study, the fact that the NS patients and control were matched for age and sex, that socio-economic background of cases and control provided the basis for further comparisons. The concerns about the cofounding effects of these factors are therefore minimal.

The present study has also shown that both folate and cyanocobalamin levels were lower in the NS than healthy children. There were significant inverse correlations between plasma homocysteine level and serum folate and vitamin B12. These are in agreement with the findings of other earlier studies in children [25–27]. Reduced nutritional intakes of vitamins and possibly other micronutrients in these children may explain the decrease in serum concentration of these essential vitamins [28]. There is a higher prevalence of folate deficiency and infectious diseases in Africans both of which impair folate assimilation[29]. These findings indicate the need for adequate folate in children's diet. This could be achieved by increasing vegetables and fruit consumption, both good sources of folate, which will reduce homocysteine levels and increase folate levels as reported in previous study [30].

Further, in this study, there was a negative correlation between homocysteine and creatinine clearance in patients with NS and healthy children. The data also showed that plasma homocysteine correlates with serum albumin as previously reported by Bostom et al.[31]. These correlations suggest that the plasma levels of homocysteine in the NS patients might depends on both creatinine clearance and serum albumin. In addition, it appears the homocysteine level was also dependent on the serum levels of B vitamins. Although the low plasma levels of vitamin B12 and B6 observed in the NS patients might certainly contributed to the development of hyperhomocysteinaemia, it is possible that other features of the NS, for example the loss of vitamin B6 protein carriers in the urine, might have influenced vitamin B6 status. The low level of vitamin B6 found in this study had been reported in some other studies of children with NS [32, 33] which demonstrated that loss of vitamin B6 protein carriers in the urine might influence vitamin B6 status.

One of the strength of the present study is the fact that estimated glomerular filtration rate of the NS patients and control was not significantly different. This suggests that the patients’ renal function were similar to healthy children who participated as the control group. Though it was difficult to establish the exact onset of the illness, information obtained from the caregivers revealed that the longest duration of symptom was about four months. It is not unlikely that many of the patients may have been ill long before the parents or caregivers sought help in health facilities. Recent review of the burden of kidney diseases in Nigeria showed that most patients present late to the hospital and they may attempt to deny it for reason of not wanting to be blamed [34]. Some of the possible implications of delay in presenting in the hospital include poor nutritional status and this might considerably affect levels of macro and micronutrients in sick children. However, considering the health implications of low levels of serum folate, vitamin B12 and vitamin B6, it is important that adequate insights are gained into the import and reasons for low vitamins in patients with nephrotic syndrome. Nevertheless, the data from this study should be viewed in the light of lack of comparable data from Nigerian children. Thus, these findings has provided basis for further study into the effects of nephrotic syndrome on plasma B vitamins and its potential usefulness in ameliorating the effects of hyperhomocysteinaemia. An important limitation to interpretation of findings from the present study is that the interplay of genetic factors and homocysteine was not assessed. Homozygosity for the methylenetetrahydrofolate reductase (MTHFR) 677C-T polymorphism is the most common genetic determinant [35]. Individuals with the (MTHFR) 677TT genotype usually have higher tHcy than those with 677CC variant [36]. Though this genetic factor was not evaluated, the results of this study still provide sufficient background for future research on plausible benefits of B vitamins supplementation. Another limitation that needs to be considered in the interpretation of data presented in this study is the small sample size. A larger number of patients were desirable but only those who presented at the study centre were recruited over a 15 month period. However, it is worth noting that the number of NS studied was statistically sufficient to power this case control study even at 90% power.

## Conclusion

Clinically important hyperhomocysteinaemia and low B vitamins occur in Nigerian children with nephrotic syndrome. This data suggest that potential usefulness of folate and vitamin B supplementation for reducing high homocysteine levels in nephrotic syndrome need to be further investigated. Findings from this study have public health importance, considering the fact that cardiovascular complications are gradually becoming a common cause of death in many developing countries especially among individuals with renal diseases [37]. With increasing survival of children suffering from NS, it is not unlikely that the risk of cardiovascular diseases may be heightened by the prevailing hyperhomocysteinaemia if the situation is not addressed early in their care.
